# Assessment of right ventricular size and function from cardiovascular magnetic resonance images using artificial intelligence

**DOI:** 10.1186/s12968-022-00861-5

**Published:** 2022-04-11

**Authors:** Shuo Wang, Daksh Chauhan, Hena Patel, Alborz amir-Khalili, Isabel Ferreira da Silva, Alireza Sojoudi, Silke Friedrich, Amita Singh, Luis Landeras, Tamari Miller, Keith Ameyaw, Akhil Narang, Keigo Kawaji, Qiang Tang, Victor Mor-Avi, Amit R. Patel

**Affiliations:** 1 Department of Medicine, University of Chicago, University of Chicago Medical Center, 5758 S. Maryland Avenue, Chicago, IL MC906760637 USA; 2 Department of Radiology, University of Chicago, Chicago, IL USA; 3 Circle Cardiovascular Imaging, Calgary, Canada; 4 Northwestern University, Chicago, IL USA; 5 Peking University Shougang Hospital, Beijing, China; 6 Illinois Institute of Technology, Chicago, IL USA

**Keywords:** Artificial intelligence, Deep learning, Right ventricular function, Right ventricular ejection fraction

## Abstract

**Background:**

Theoretically, artificial intelligence can provide an accurate automatic solution to measure right ventricular (RV) ejection fraction (RVEF) from cardiovascular magnetic resonance (CMR) images, despite the complex RV geometry. However, in our recent study, commercially available deep learning (DL) algorithms for RVEF quantification performed poorly in some patients. The current study was designed to test the hypothesis that quantification of RV function could be improved in these patients by using more diverse CMR datasets in addition to domain-specific quantitative performance evaluation metrics during the cross-validation phase of DL algorithm development.

**Methods:**

We identified 100 patients from our prior study who had the largest differences between manually measured and automated RVEF values. Automated RVEF measurements were performed using the original version of the algorithm (DL1), an updated version (DL2) developed from a dataset that included a wider range of RV pathology and validated using multiple domain-specific quantitative performance evaluation metrics, and conventional methodology performed by a core laboratory (CORE). Each of the DL-RVEF approaches was compared against CORE-RVEF reference values using linear regression and Bland–Altman analyses. Additionally, RVEF values were classified into 3 categories: ≤ 35%, 35–50%, and ≥ 50%. Agreement between RVEF classifications made by the DL approaches and the CORE measurements was tested.

**Results:**

CORE-RVEF and DL-RVEFs were obtained in all patients (feasibility of 100%). DL2-RVEF correlated with CORE-RVEF better than DL1-RVEF (r = 0.87 vs. r = 0.42), with narrower limits of agreement. As a result, DL2 algorithm also showed increasing accuracy from 0.53 to 0.80 for categorizing RV function.

**Conclusions:**

The use of a new DL algorithm cross-validated on a dataset with a wide range of RV pathology using multiple domain-specific metrics resulted in a considerable improvement in the accuracy of automated RVEF measurements. This improvement was demonstrated in patients whose images were the most challenging and resulted in the largest RVEF errors. These findings underscore the critical importance of this strategy in the development of DL approaches for automated CMR measurements.

## Introduction

Right ventricular (RV) function is an important predictor of outcomes in patients with heart disease [1–3]. However, the non-invasive assessment of RV size and function is significantly limited by its complex geometry. Cardiovascular magnetic resonance (CMR) quantification of RV volume using the method of disks is currently considered the reference standard for RV size and RVejection fraction (RVEF), because the entire chamber is imaged, the boundary between the RV myocardium and blood pool is clearly delineated, and no geometric assumptions about the RV shape are needed [4]. Indeed, the assessment of RV size and function using CMR has been shown to be of prognostic significance in patients with ischemic [5] and non-ischemic cardiomyopathy [6], valvular heart disease [7], congenital heart disease [8], and pulmonary hypertension [9]. Furthermore, quantification of RV size is an important determinant for guiding interventions, such as pulmonary valve replacement in patients with tetralogy of Fallot [10]. In patients with ischemic or non-ischemic cardiomyopathy, the presence of reduced RVEF in addition to reduced left ventricular (LV) function can better identify patients who may benefit from implantable cardioverter defibrillator and other interventions [1, 2].

Nevertheless, manual segmentation of the RV cavity is time-consuming and has greater inter-reader variability than is seen for the LV cavity [11–14]. The availability of an image analysis tool that can accurately and automatically quantify RV size and function from CMR images would not only help standardize image interpretation, but also improve clinical workflow. While several commercial tools are capable of accurately assessing LV volumes and ejection fraction, it is well recognized that these tools do not perform as well for the RV assessment. Indeed, we recently demonstrated that the RVEF measured using several commercial software tools resulted in clinically significant errors when compared to conventional measurements performed by an expert [15].

Most currently available software that automatically quantifies RV size and function from CMR images are complex algorithms that use a form of artificial intelligence referred to as deep learning (DL) [16, 17]. Such algorithms are typically trained and tested using thousands of CMR images with the key anatomy annotated by expert physicians. Due to the scarcity of well annotated CMR images, publicly available datasets which are fairly homogenous in nature, are often utilized to develop these DL algorithms. Although a homogenous dataset may be a good way to build the foundations of a DL algorithm, as it often implies standardized labelling or contouring procedures, the absence of a heterogeneous dataset during the testing phase of development may result in an algorithm that functions poorly in clinical practice, where more diverse disease states and image quality are encountered that were not adequately reflected by the training dataset. In this study, we hypothesized that the use of more diverse CMR datasets, which incorporate a wider range of RV pathology, scanner vendors, and imaging quality in addition to the use of multiple domain-specific quantitative performance evaluation metrics during cross-validation phase of DL algorithm development would result in a more generalized model and thus more accurate quantification of RV size and function in a subset of patients in whom the previous version of the software did not perform well.

## Methods

Data and materials used in this study will not be made publicly available.

### Population and study design

From our prior study [15], in which we compared the ability of several commercial DL-algorithms to automatically calculate RVEF against reference measurements made by a clinical expert. For this study, we selected 100 cases (of the 200 total) with the largest discrepancies between the DL-generated and reference RVEFs (Group 1). Automated RVEF measurements were performed using an updated version of the DL algorithm (DL2) cross-validated on a diverse dataset of CMR images. To assess the improvement in algorithm performance afforded by this strategy, these measurements were compared side-by-side with the measurements performed using the original software (DL1) against the same reference standard. These comparisons included actual RVEF values and also classification of RV function based on these values, namely normal, mildly to moderately reduced and severely reduced function. In addition, similar comparisons were performed on the remaining 100 patients in whom the original DL1 algorithm performed well, i.e. with the least discrepancies with the reference RVEFs (Group 2), in order to verify that the algorithm modifications did not have detrimental effects on RVEF measurements in these patients.

All subjects had previously signed informed consent to be included in a CMR registry permitting their images, clinical data, and outcomes to be used for future research. Patient demographics and clinical history were extracted from the electronic medical records. The protocol was approved by the Institution Review Board.

### Deep learning algorithm development

#### DL algorithm 1

The DL1 segmentation model, included in cvi42 (version 5.11, Circle Cardiovascular Imaging, Calgary, Alberta, Canada), is founded on a convolutional neural network (CNN) based on the U-net architecture. The CNN is trained to associate pixel intensities of a CMR image to segmentation maps corresponding to the desired ventricular contours. During the training stage, the model parameters of CNN were optimized to reduce an energy function computed using the pixel-wise cross-entropy loss function, which penalizes the CNN when it does not correctly predict the segmentation label of a given pixel.

The initial machine learning-based RV contouring CNN was trained on multiple datasets summarized in Table 1. Standard image augmentation techniques were employed during the training phase. The training hyper-parameters of the DL1 model were selected by training multiple models with varying hyperparameters and selecting the model that performed the best on the cross-validation data presented in Table 1, which comprised solely of United Kingdom Biobank (UKBB) studies. The candidate models were compared against one another by computing the mean dice similarity coefficient (DSC) between predicted contours and manual annotations for each contour (RV, LV endocardium, and LV epicardium) independently.

**Table 1:** Summary of data (total number of cardiovascular magnetic resonance (CMR) images) used during the training and cross-validation phase of the development of the deep learning (DL) algorithms described herein

	Training datasets			Cross-validation datasets		
Datasets	UKBB	ToF	Clinical	UKBB	ToF	Clinical
DL1	67,907	513	1900	16,977	0	0
DL2	67,907	513	2033	26,179	460	9612

Three datasets were used during algorithm training consisting of images from the UK Biobank (UKBB), tetralogy of Fallot (ToF), and clinical dataset consisting of various pathologies including hypertrophic cardiomyopathy, dilated cardiomyopathy, pulmonary hypertension, and left ventricular non-compaction cardiomyopathy

#### DL algorithm 2

DL2, included in cvi42 (Version 5.13, Circle Cardiovascular Imaging) was trained using the exact same CNN architecture as DL1. Prior to training DL2, 9745 new images were added to the Clinical dataset and the dataset was resampled such that roughly 20% of the cases were used in training and the remainder were kept for cross-validation. 460 additional tetralogy of Fallot images and 9202 additional images from UKBB were also added to the cross-validation dataset. Importantly, the cross-validation process was performed on this dataset and model selection was done in a manner that was blind to the cases on which it was tested and reported on in this study.

Additional quantitative performance metrics were used to assess the performance of the algorithm during the cross-validation phase as a spatial overlap metric, such as DSC fails to capture potential biases in the predicted contours [18]. In addition to DSC, the contour spatial distance based Hausdorff distance metric and domain specific performance metrics were computed, including absolute errors in RV volume prediction, LV volume and LV ejection fraction (LVEF) prediction, myocardial mass consistency and contour consistency. The total number of discontinuous predictions in the midventricular region was also computed to favor the selection of models with highly consistent predictions. The mean and standard deviation of all performance metrics were considered independently to select the most performant model. The change in the cross-validation process resulted in a different choice in hyperparameters. Specifically, there were changes in how each training batch was sampled, the frequency of the augmentations, and the point in time at which the training algorithm is stopped.

### Image acquisition

Vasodilator contrast-enhanced stress perfusion CMR imaging was performed using a 1.5-T scanner (Achieva, Philips Healthcare, Best, The Netherlands) with a five-element phased array cardiac coil. Retrospectively gated cine images were obtained following stress perfusion imaging using a balanced steady-state free precession (bSSFP) sequence, during approximately 5-s breath holds (repetition time 2.9 ms; echo time 1.5 ms; flip angle 60°; temporal resolution 30–40 ms). Standard long-axis views were obtained, including four-chamber, two-chamber, and three-chamber images. In addition, six to ten short-axis slices were obtained from the LV and RV base to the apex (slice thickness 8 mm; gap 2 mm).

### Conventional image analysis

The cine-CMR images were analyzed semi-automatically using commercial software (CardioAI, Arterys, San Francisco, California, USA) by an experienced expert reader in our core lab, who was blinded to clinical information. Using the short-axis cine images, the RV endocardial boundary was automatically detected by the software and then corrected by the expert in end-diastolic and end-systolic frames. RV papillary muscles and trabecular tissue were included in the blood pool volume. The RV outflow tract was included in the RV volume to the level of the pulmonary valve. When the boundary between the atrium and ventricle was unclear, long axis views and other frames of the cardiac cycle were reviewed to differentiate between the two chambers. Simpson’s method of disks was used to calculate RV end-diastolic volume (RVEDV) and end-systolic volume (RVESV), and RVEF. All volume measurements were indexed to body surface area. The RV measurements made by the core lab expert were considered as the reference standard (CORE-RVEF, CORE-RVEDV, CORE-RVESV). CORE-RVEF were used to divide the patient cohort into three clinically relevant groups [19–21]: severely reduced RVEF (≤ 35%), mildly to moderately reduced RVEF (35–50%), and normal RVEF (≥ 50%).

### DL image analysis

The RVEF was determined using two different fully automated DL-algorithms DL1 and DL2, described above. Cine images were uploaded into the software and fully automated segmentation was then performed without any further user input. A plane demarcating the ventricular base was identified automatically. Using the entire short axis stack and standard long axis views, both DL1 and DL2 generated time-volume curves, from which end-diastole and end-systole were automatically determined and used to calculate RVEDV, RVESV, and RVEF.

### Statistics

Continuous variables were tested for normal distribution. Continuous variables that were not normally distributed, were presented as the median with interquartile range (IQR). Categorical variables were presented as absolute numbers with percentages. Inter-technique comparisons included linear regression analysis with Pearson correlation coefficients and Bland–Altman analyses of biases and limits of agreement. This included the agreement between each of the DL techniques (DL1 and DL2) against the CORE reference. Histograms displaying the difference in RVEF measurements between the DL algorithms and the core lab were generated. Confusion matrices were generated for each DL-RVEF algorithm to display the concordance/discordance with the CORE-RVEF for each of the RVEF categories (≤ 35%, 35–50%, and ≥ 50%). The sensitivity, specificity, and accuracy of DL1-RVEF and DL2-RVEF algorithms’ ability to correctly categorize RV function were also calculated. Reproducibility was tested for the 2 DL algorithms on 20 randomly selected patients and by manual analyses by two expert readers, including inter- and intra-observer reproducibility. Inter- and intra- observer variability was assessed using intraclass correlation coefficients (ICC) and coefficients of variation (CoV). P-values < 0.05 were considered significant. Analyses were performed using SPSS software (version 23.0, Statistical Package for the Social Sciences, International Business Machines, Inc., Armonk, New York, llinois, USA).

## Results

### Patient demographics

**Fig. 1:**
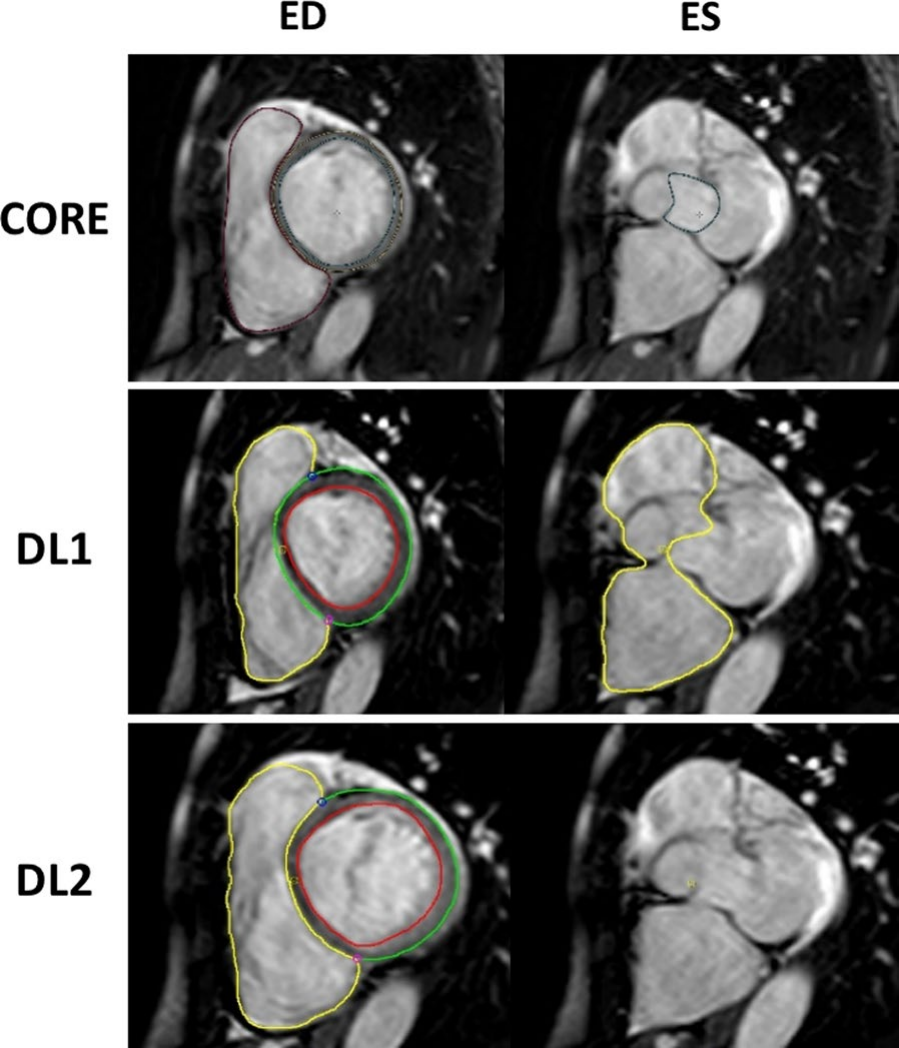
Examples of images of contours detected by the core lab (CORE), original deep learning (DL1) and updated deep learning (DL2) (shown from top to bottom). Non-ischemic cardiomyopathy at basal slice

Patient characteristics for Group 1 are shown in Table 2 along with the relevant imaging findings. Figure 1 shows examples of RV boundaries as determined by the core-lab and by the 2 DL algorithms.

**Table 2:** Population baseline characteristics and CMR parameters in the clinical setting

Parameters	Median (interquartile range) or n (%)
Clinical	Overall (n = 100)
Gender, male	41 (41%)
Age, years	61 (53–69)
Body mass index, kg/m^2^	28 (24–32)
Body surface area, m^2^	1.9 (1.8–2.1)
Race, %	
Black	42 (42%)
White	44 (44%)
Hispanic	7 (7%)
Asian	5 (5%)
Unknown	2 (2%)
Diagnosis, n (%)	
Coronary artery disease	56 (56%)
Hypertension	81 (81%)
Diabetes	42 (42%)
Post-heart transplant	5 (5%)
Post-CABG	19 (19%)
Congenital heart disease	2 (2%)
Pulmonary hypertension	7 (7%)
Chronic lung disease	25 (25%)
Obstructive sleep apnea	14 (14%)
Cardiomyopathy	39 (39%)
ICM	16 (16%)
NICM	23 (23%)
CMR	
LVEDV, ml	157 (123–205)
LVEDVI, ml/m^2^	80 (67–105)
LVESV, ml	80 (52–125)
LVESVI, ml/m^2^	40 (27–63)
LVM, g	110 (88–129)
LVMI, g/m^2^	56 (46–66)
LVEF, %	49 (39–61)
RVEDV, ml	149 (116–173)
RVEDVI, ml/m^2^	75 (62–89)
RVESV, ml	69 (50–95)
RVESVI, ml/m^2^	35 (26–49)
RVEF, %	54 (45–58)
LGE, n (%)	43 (43%)
Ischemic pattern	29 (29%)
Non-ischemic pattern	10 (10%)
Both patterns	4 (4%)

*ICM* ischemic cardiomyopathy, *LGE* late gadolinium enhancement, *LVEDV* left ventricular end-diastolic volume, *LVEDVI* left ventricular end-diastolic volume index, *LVEF* left ventricular ejection fraction, *LVESV* left ventricular end-systolic volume, *LVESVI* left ventricular end-systolic volume index, *LVM* left ventricular mass, *LVMI* left ventricular mass index, *NICM* non-ischemic cardiomyopathy, *RVEDV* right ventricular end-diastolic volume, *RVEDVI* right ventricular end-diastolic volume index, *RVEF* right ventricular ejection fraction, *RVESV* right ventricular end-systolic volume, *RVESVI* right ventricular end-systolic volume index

### Relationship between CORE-RVEF and DL-RVEF

**Fig. 2:**
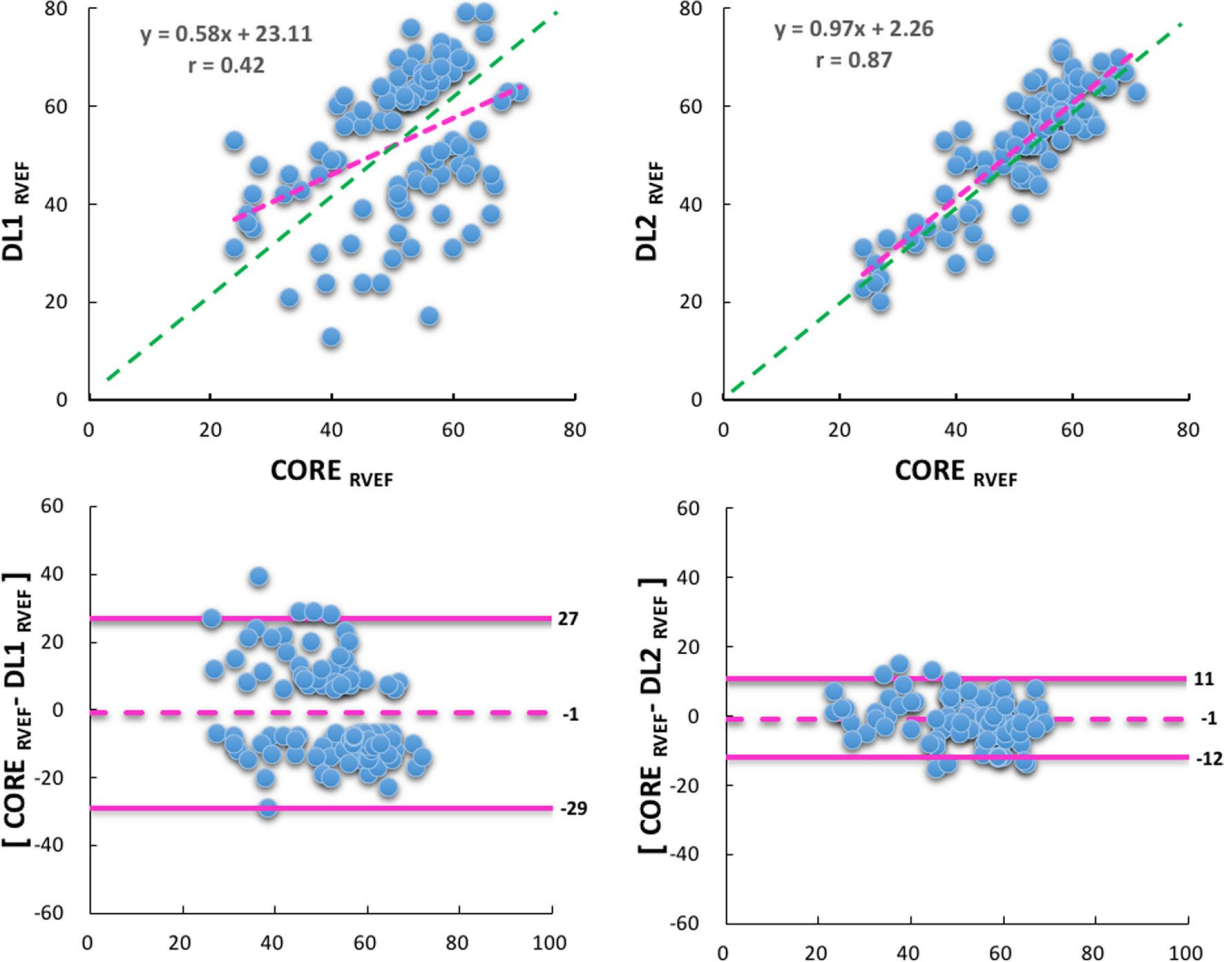
Linear regression plots (top) and Bland–Altman plots (bottom) comparing CORE and DL1 (left), CORE and DL2 (right). Red lines represent the regression lines, and green lines represent perfect agreement (unity lines)

**Fig. 3:**
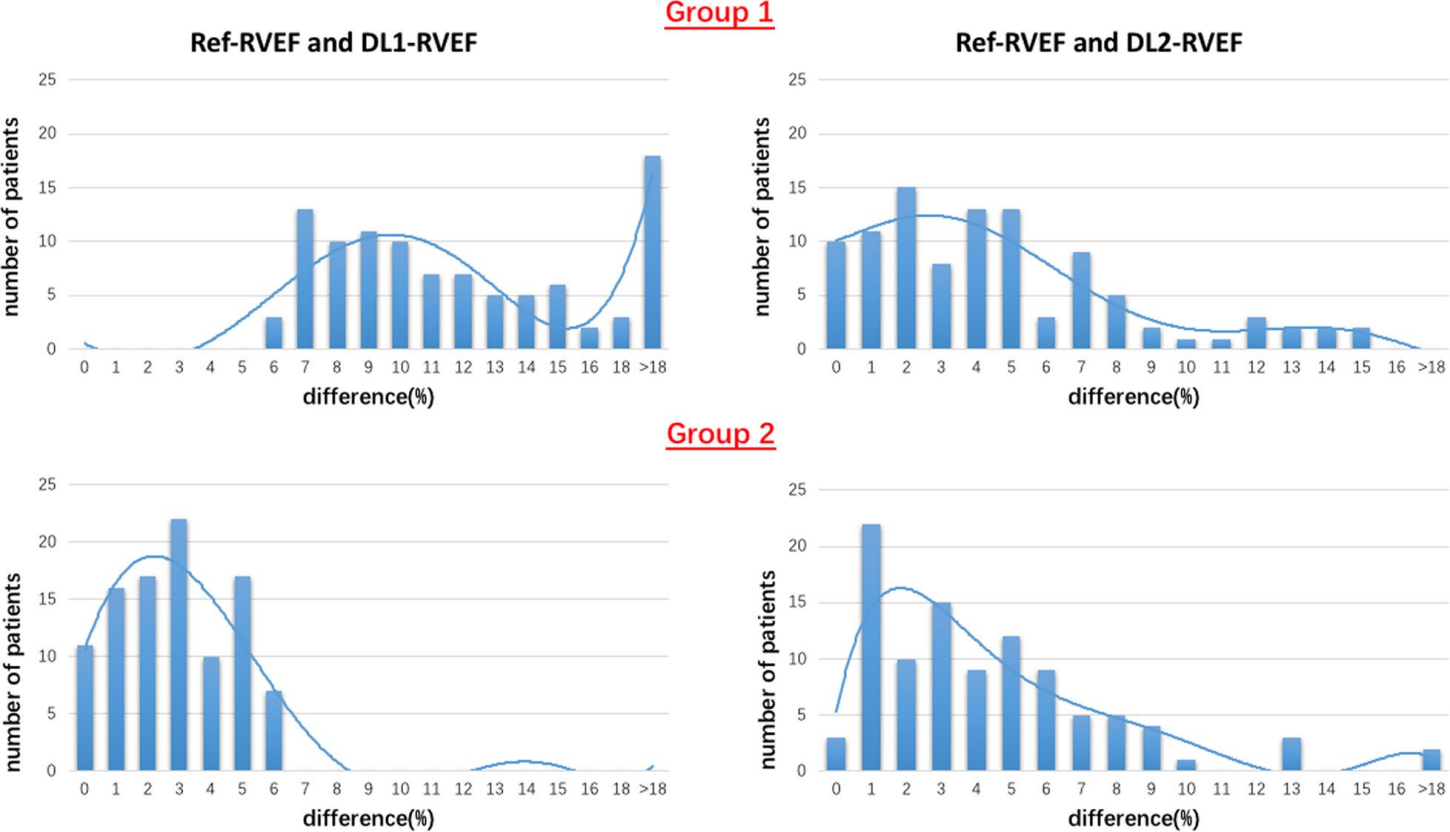
Histograms showing the distribution of absolute differences between CORE right ventricular ejection fraction (RVEF) versus DL1 RVEF (left) and CORE RVEF versus DL2 RVEF (right) for Group 1 (top) and Group 2 (bottom). See text for details

Compared to CORE-RVEF, the correlations of DL1-RVEF and DL2-RVEF were 0.42 and 0.87, respectively (Fig. 2). The results of Bland–Altman analyses for the two DL algorithms in comparison to the core lab analysis are depicted in Fig. 2. Algorithm DL2 performed significantly better than DL1, as reflected by considerably narrower limits of agreement: − 1 ± 11% for DL2 versus − 1 ± 28% for DL1. Figure 3 shows histograms that demonstrate the frequency of absolute differences between CORE-RVEF and each of the DL-RVEF algorithms for Group 1 (top) and Group 2 (bottom). For DL1, no case in Group 1 had ≤ 5% error as a consequence of the study design, while errors > 10% were noted in 35(35%) of cases and errors exceeding 20% were present in 17(17%). In contrast, for algorithm DL2, 44 (44%) cases in Group 1 were within 3% absolute error and 70(70%) cases were within 5% error, while only 10 (10%) cases showed errors > 10% when compared to CORE-RVEF. In group 2, no case had > 5% error according to DL1, as a consequence of the study design, and DL2 resulted in a relatively small number of cases with larger errors.

### Relationship between CORE-RV size and DL-RV size

**Fig. 4:**
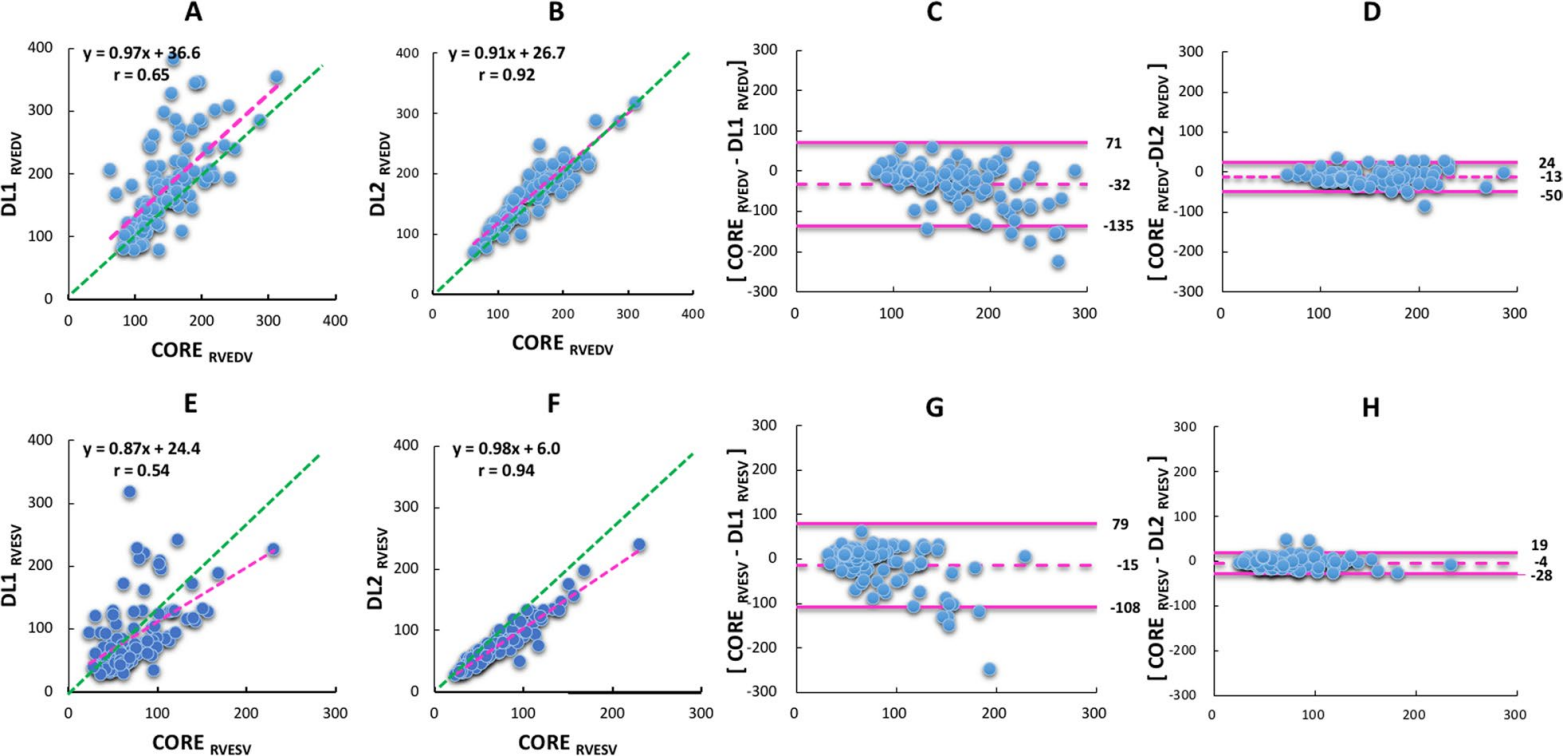
Linear regression plots (left) comparing CORE RVEDV and DL1 RVEDV (**A**), CORE RVEDV and DL2 RVEDV (**B**), CORE RVESV and DL1 RVESV (**E**), CORE RVESV and DL2 RVESV (**F**). Bland–Altman plots (right) comparing CORE RVEDV and DL1 RVEDV (**C**), CORE RVEDV and DL2 RVEDV (**D**), CORE RVESV and DL1 RVESV (**G**), CORE RVESV and DL2 RVESV (**H**). Red lines represent the regression lines, and green lines represent perfect agreement (unity lines). RVEDV, right ventricular end-diastolic volume; RVESV, right ventricular end-systolic volume

For RVEDV and RVESV, algorithm DL1 was less accurate than DL2 when compared to CORE lab reference values (Fig. 4). For RVEDV, the correlations between CORE-RVEDV and DL-RVEDV were 0.65 (DL1) and 0.92 (DL2). For RVESV, the correlations between CORE-RVESV and DL-RVESV were 0.54 (DL1) and 0.94 (DL2). Bland–Altman analyses of comparison between CORE-RVEDV and DL-RVEDV resulted in smaller biases and narrower limits of agreement for DL2: − 32 ± 103 ml (DL1), − 13 ± 37 ml (DL2). For RVESV, the bias and limits of agreement were − 15 ± 93 ml (DL1), − 4 ± 24 ml (DL2).

### Reproducibility

The intraclass correlations for the inter- and intra-observer variability of manual measurements were 0.90 and 0.94 for the RVEF, 0.94 and 0.96 for the RVEDV, 0.94 and 0.97 for the RVESV, respectively. In contrast, DL algorithms showed zero variability in all repeated measurements, due to their fully-automated, deterministic nature.

### Classification into ejection fraction categories

**Fig. 5:**
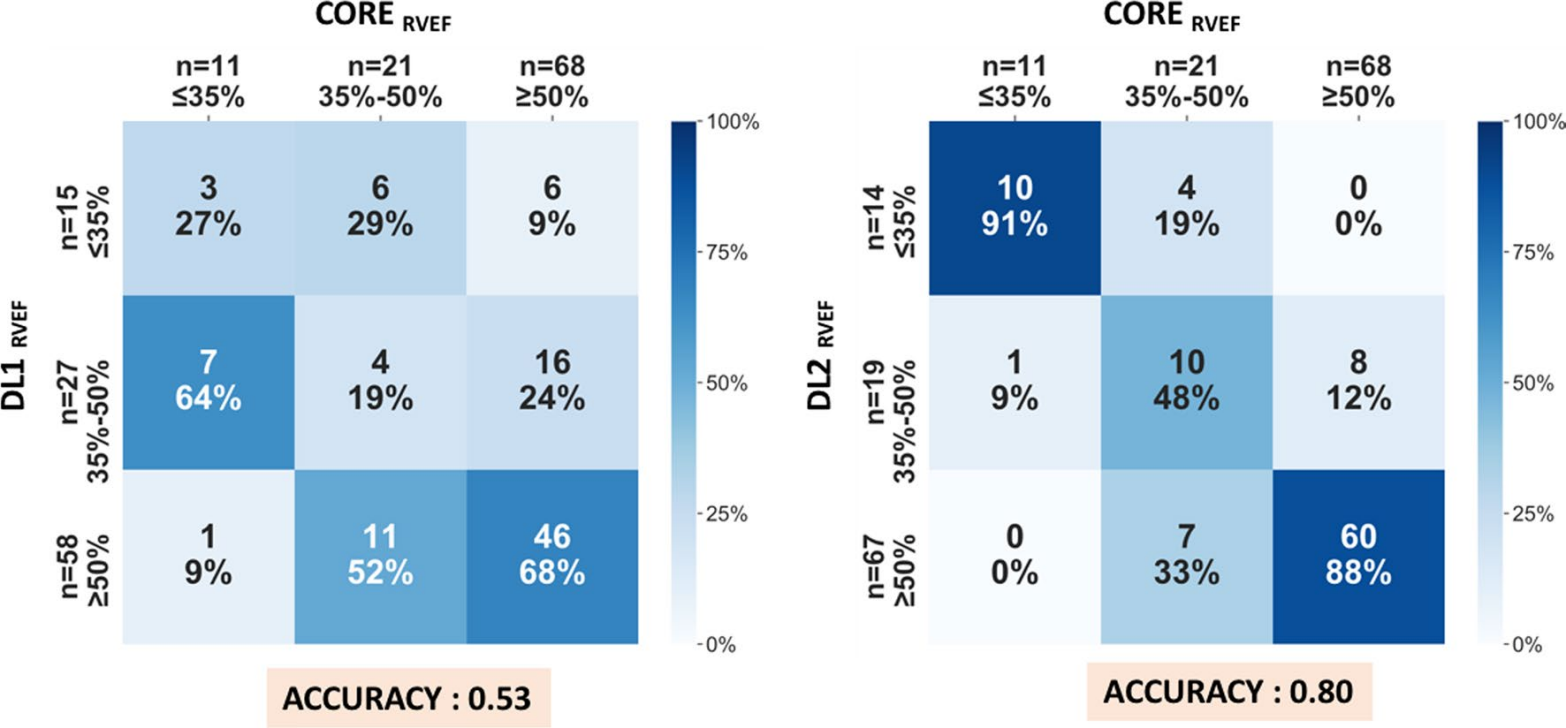
Confusion matrices showing accuracy of DL-RVEF to correctly categorize into clinically meaningful RVEF groups as defined by the RVEF by core lab (CORE-RVEF). Across true label rows, the numbers in the boxes represent the number and percentage of labels classified for each group. Color intensity corresponds to percentage, see heat map on the right

Based on CORE-RVEF, 11 of the 100 patients had severely reduced RVEF (≤ 35%), 21 had mildly to moderately reduced RVEF (35–50%), and 68 had preserved RVEF (≥ 50%) (Table 3). The accuracy of the DL1 and DL2 algorithms for classifying RV function into appropriate EF group was 0.53 and 0.80, respectively. The lowest rates of accurate classifications were noted in the middle RVEF category of 35–50% (Fig. 5).

**Table 3:** Population characteristics and imaging parameters in subgroups

Parameters	Median (interquartile range) or n (%)		
Clinical	RVEF ≤ 35% (n = 11)	RVEF35-50% (n = 21)	RVEF ≥ 50% (n = 68)
Gender, male	6 (55%)	15 (71%)	38 (56%)
Age, years	59 (51–80)	63 (50–69)	61 (54–69)
Body mass index, kg/m^2^	29 (25–38)	26 (23–31)	29(25–32)
Body surface area, m^2^	2.0 (1.8–2.4)	1.9 (1.7–2.2)	2.0 (1.8–2.1)
Race, %			
Black	8 (73%)	9 (43%)	25 (37%)
White	2 (18%)	11 (52%)	31 (46%)
Hispanic	1 (9%)	0 (0)	6 (9%)
Asian	0 (0)	0 (0)	5 (7%)
Unknown	0 (0)	1 (5%)	1 (2%)
Diagnosis, n (%)			
Coronary artery disease	4 (36%)	10 (48%)	42 (62%)
Hypertension	8 (73%)	17 (81%)	56 (82%)
Diabetes	6 (55%)	7 (33%)	29 (43%)
Post-heart transplant	0 (0)	1 (5%)	4 (6%)
Post-CABG	2 (18%)	2 (10%)	15 (22%)
Congenital heart disease	0 (0)	0 (0)	2 (3%)
Pulmonary hypertension	1 (9%)	3 (14%)	3 (4%)
Chronic lung disease	0(0)^●^	11 (52%)^#^	14 (21%)
OSA	1 (9%)	1(5%)	12 (18%)
Cardiomyopathy	10 (91%)*	12 (57%)^#^	17 (25%)
ICM	4 (36%)*	4 (19%)	8 (12%)
NICM	6 (55%)*	8 (38%)^#^	9 (13%)
CMR			
LVEDV, ml	238 (179–310)*	166 (136–218)	147 (116–192)
LV DVI, ml/m^2^	124 (85–139)*	97 (74–124)^#^	71 (64–97)
LVESV, ml	176 (128–234)^●^*	111 (72–134)^#^	65 (47–100)
LVESVI, ml/m^2^	93 (62–105)^●^*	55 (38–75) ^#^	32 (24–52)
LVM, g	115 (103–226) *	125 (107–140)^#^	101 (83–122)
LVMI, g/m^2^	62 (54–93)*	66 (54–76)^#^	52 (44–61)
LVEF, %	27 (22–30)^●^*	43 (36–47)^#^	54 (44–63)
RVEDV, ml	190 (148–224)*	164 (124–191)	134 (113–164)
RVEDVI, ml/m^2^	87 (74–115)*	85 (74–95)^#^	71 (61–81)
RVESV, ml	131 (108–161)*	95 (75–112)^#^	59 (46–73)
RESVI, ml/m^2^	65 (54–77)^●^*	50 (42–54)^#^	30 (25–36)
RV EF, %	27 (26–32)^●^*	43 (40–46)^#^	58 (53–61)
LGE, n (%)	8 (73%)^●^*	9(43%)	26 (38%)
Ischemic pattern	1 (9%)^●^*	6 (29%)	22 (32%)
Non-ischemic pattern	5 (46%)*	2 (10%)	3 (4%)
Both patterns	2 (18%)	1 (5%)	1 (2%)

*ICM* ischemic cardiomyopathy, *LGE* late gadolinium enhancement, *LVEDV* left ventricular end-diastolic volume, *LVEDVI* left ventricular end-diastolic volume index, *LVEF* left ventricular ejection fraction, *LVESV* left ventricular end-systolic volume, *LVESVI* left ventricular end-systolic volume index, *LVM* left ventricular mass, *LVMI* left ventricular mass index, *NICM* non-ischemic cardiomyopathy, *RVEDV* right ventricular end-diastolic volume, *RVEDVI* right ventricular end-diastolic volume index, *RVEF* right ventricular ejection fraction, *RVESV* right ventricular end-systolic volume, *RVESVI* right ventricular end-systolic volume index

●P < 0.05, RVEF ≤ 35% and RVEF 35–50%

^*^P < 0.05, RVEF ≤ 35% and RVEF ≥ 50%

^#^P < 0.05, RVEF 35–50% and RVEF ≥ 50%

## Discussion

In this study, we aimed to determine the impact of including CMR images with diverse RV pathology during the cross-validation stage on the performance of a DL algorithm developed to automatically quantify RV size and function. We found that such an approach resulted in substantial and clinically important improvements in the performance of the algorithm in patients whose images were difficult to handle for the previous version of the algorithm. These findings were in agreement with several previous studies [22–24].

RV dysfunction is associated with poor clinical outcomes in a wide range of cardiovascular disorders including but not limited to acute myocardial infarction, LV heart failure, congenital heart disease, arrhythmogenic cardiomyopathy, and pulmonary hypertension [25, 26]. RV systolic function is highly sensitive to changes in the afterload with small increases in afterload causing large decreases in stroke volume. Importantly, left heart disease can affect RV function through abnormal motion of the ventricular septum. Thus, quantification of RVEF, as a biomarker of RV systolic function, can be used to guide therapeutic decision making and to assess prognosis [27–29]. Although RVEF is a continuous variable, RVEF cutoff values have been shown to have prognostic value in patients with dilated cardiomyopathy [28] and in other disease states [9]. The importance of RVEF for prediction of cardiovascular outcomes was demonstrated in a cohort comprised of a broad range of cardiovascular diseases by demonstrating that each 10% drop in RVEF was associated with a 1.33-fold increased risk [3]. In fact, RVEF is associated with poor outcomes even in individuals with a preserved LVEF [3, 30].

In recognition of the importance of RV function, CMR is increasingly being used to assess the RV due to its ability to accurately quantify chamber size and function without being limited by the complex RV geometry. However, the process remains time consuming and even fully automated, commercially available DL techniques used to quantify RVEF may perform poorly in some patients, and as a result may fail to predict clinical events [31], suggesting that the current approach to developing these algorithms needs to be refined. In our study, we investigated a new approach to selecting a candidate algorithm to be used in clinical practice. The biggest source of improvement in this study is from the increased variability in the cross-validation dataset and addition of multiple performance metrics employed to guide model selection. The addition of 10,161 images to include RV pathologies, such as pulmonary arterial hypertension, repaired tetralogy of Fallot, etc. were used to revamp the cross-validation strategy. Contour overlap metrics and the ability of accurate clinical biomarkers and results were factored into the exploration of DL2 algorithm. Additionally, the strict enforcement of a spatiotemporal consistency in the predicted outputs was adopted to post-processing in all stacks, which ensured the removal of outliers in the event that contours are predicted above the basal slice or below the apex.

Although other studies have examined the accuracy of DL algorithms to quantify RVEF in comparison to a human expert [12, 32, 33], they did not examine the clinical implications of any specific sources of error. Rahman et al., reported high levels of agreement between the DL algorithm and manual measurement of RVEF, but the focus was limited to the healthy population [34]. In a study by Hakim et al., when comparing automated RVEF measurements to a human expert generated reference values, the DL algorithm showed an r-value of 0.76. The major source of error has typically been explained by poor automated segmentation at the basal slice, as was observed in prior studies [35]. In our prior study [15], we compared manual RVEF by a clinician to automated RVEF measurements made by three DL methods developed using publicly available CMR datasets and found that all three DL methods performed relatively poorly in a significant proportion of the patients.

**Fig. 6:**
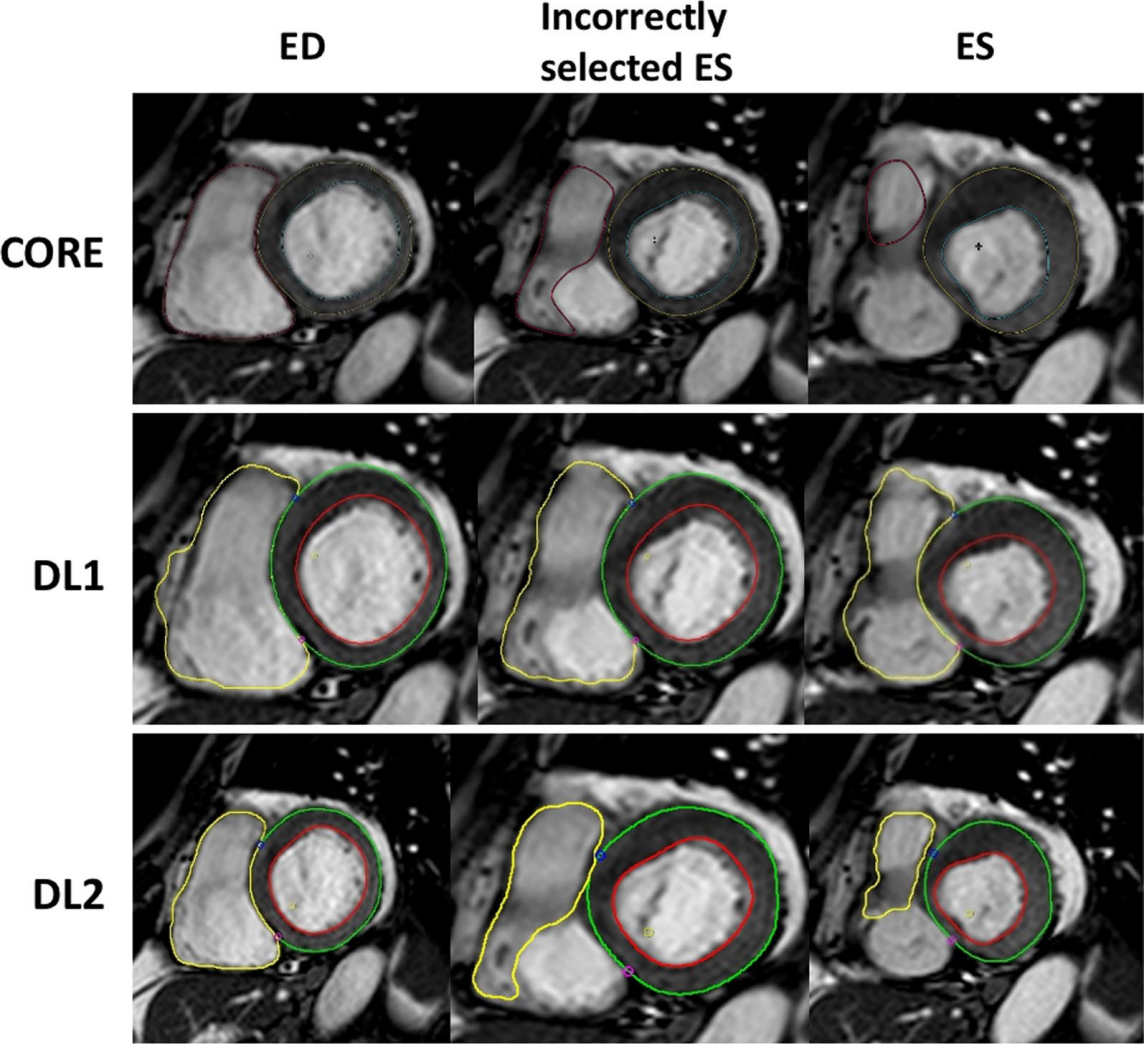
Example of images with contours detected by CORE, DL1 and DL2 algorithms. CORE and DL2 identified the correct ED and ES phases, but DL1 identified an incorrect ES phase. The CORE-RVEF was 52%, DL1-RVEF was 24%, and DL2-RVEF was 50%

In this current study, we selected from our prior study [15] the 100 cases, in which the DL algorithm performed the worst, and found that an algorithm developed using a new approach with enhanced cross-validation resulted in considerable improvement in its clinical performance. Indeed, measurements of RVEDV, RVESV, and RVEF made using the improved algorithm correlated better with the reference measurements. This increased correlation translated into more accurate classification of RVEF function. However, it is important to note that the correlation between the core laboratory measurement and the DL algorithm was not perfect, 10% of cases still had a > 10% absolute error in the RVEF measurement, and 20% of patients were still categorized into an incorrect RVEF group. This emphasizes the need for additional algorithmic improvements in the future. Detecting the RV endocardial contour is challenging for both the clinician and automated DL algorithms. This can be attributed to the unique morphology, thinner myocardium, and higher trabeculation burden of the RV chamber. The basal short-axis slice is most often inaccurately segmented by both an expert and by the DL algorithms, particularly at the boundary of the pulmonary artery and right atrium. Furthermore, the RV apex is frequently incorrectly identified by the DL algorithms in cases of pathological RV morphology. Another challenge for the DL-algorithms for calculating RVEF is the accurate identification of the ED and ES frames (Fig. 6). In fact, some algorithms require end-diastole and end-systole to be pre-selected to overcome this challenge [36].

## Limitations

This was a single-center study performed in a relatively small number of patients. Although we demonstrated considerable improvement of algorithm performance in our cohort, it is possible that it may not perform as well on images acquired using other scanners or other CMR image acquisition techniques. Additionally, our study cohort was selected from patients referred for a CMR stress test and was not enriched with patients known to have more complex RV anatomy. Thus, it is unknown how well this current algorithm would perform when it encounters more complex clinical cases, such as cyanotic congenital heart disease and other distorted anatomies. Also, one could view as a limitation the fact that this study was performed on stress rather than resting CMR images. There were three reasons for this choice: (1) vasodilator stress is a common indication for CMR imaging; (2) we suspected that the algorithms may not perform as well in this situation, since the images used to quantify EF are usually acquired after the administration of a gadolinium-based contrast agent; and (3) the previous study, in which the original algorithm did not perform well in this subset of patients, was performed on their stress CMR images, and therefore testing the hypothesis that the retrained algorithm would perform better had to be done on the same images.

## Conclusions

The use of a diverse dataset during the cross-validation phase of DL algorithm development resulted in a considerable improvement in the accuracy of the automated analysis of RV volumes and RVEF in patients in whom the previous version of the software did not perform well. However, despite the improvement, the current fully automated algorithm is still prone to errors when compared to a core laboratory measurement due to intrinsic anatomical challenges and therefore requires verification by an experienced reader.

## Data Availability

Data supporting the results reported in the manuscript can be found in a computer workstation in the Cardiac Imaging Laboratory at the University of Chicago.

## Abbreviations

bSSFP: Steady-state free precession
CMR: Cardiovascular magnetic resonance
CNN: Convolutional neural network
CORE: Core lab
CoV: Coefficient of variation
DL: Deep learning
DSC: Dice similarity coefficient
EDV: End-diastolic volume
EF: Ejection fraction
ESV: End-systolic volume
ICC: Intraclass correlation coefficients
LV: Left ventricle/left ventricular
LVEF: Left ventricular ejection fraction
RV: Right ventricle/right ventricular
RVEDV: Right ventricular end-diastolic volume
RVEF: Right ventricular ejection fraction
RVESV: Right ventricular end-systolic volume
UKBB: United Kingdom Biobank
